# Pre-Statistical Harmonization of Behavioral Instruments Across Eight Surveys and Trials

**DOI:** 10.1093/geroni/igab046.286

**Published:** 2021-12-17

**Authors:** Diefei Chen, Eric Jutkowitz, Skylar Iosepovici, John Lin, Alden Gross

**Affiliations:** 1 Johns Hopkins Bloomberg School of Public Health, Baltimore, Maryland, United States; 2 Brown University, Brown University, Rhode Island, United States; 3 Brown University School of Public Health, Providence, Rhode Island, United States; 4 Brown University, Sugar Land, Texas, United States

## Abstract

Data harmonization methods facilitate further use of existing studies and research resources. Most statistical harmonization methods require pooling data across studies, which is complex and requires careful scrutiny of source data. Most methods (e.g., item response theory) require datasets to have common items for linking a common construct across studies: this necessitates the qualitative process of pre-statistical harmonization. Here, we document pre-statistical harmonization of items measuring behavioral and psychological symptoms (e.g., agitation, wandering, etc.) which represent problematic behaviors among people with dementia administered in a national survey (ADAMS), evaluations conducted at Alzheimer’s Disease Research Centers (NACC), and in six randomized trials (COPE, TAP, ALZQOL, ACT, REACH, ADSPlus). We describe our approach to review question content and scoring procedures to establish comparability across items prior to data pooling. We identified 327 items from 15 instruments across these eight studies. We found considerable cross-study heterogeneity in administration and coding procedures for items that measure the same domain. For example, eight items were coded as count variables in some studies but as categorical variables in others. Moreover, of the 359 items, 191 are conditionally dependent on values of another item. These issues around item response heterogeneity and conditional dependency needed to be resolved prior to estimation of item response theory models for statistical co-calibration. We leveraged several rigorous data transformation procedures to address these issues, including re-coding and winsorization. This study provides guidelines for how future research may acknowledge and address similar issues in pooling behavioral and related instruments.

